# Data on vibrational spectra of the langasites Ln_3_CrGe_3_Be_2_O_14_ (Ln = La, Pr, Nd) and *ab initio* calculations

**DOI:** 10.1016/j.dib.2019.104889

**Published:** 2019-12-16

**Authors:** Nikolay N. Kuzmin, Sergey A. Klimin, Boris N. Mavrin, Kirill N. Boldyrev, Vladimir A. Chernyshev, Boris V. Mill, Marina N. Popova

**Affiliations:** a Institute of Spectroscopy, Russian Academy of Sciences, 108840, Troitsk, Moscow, Russia; b Department of Basic and Applied Physics, Ural Federal University, 620002, Ekaterinburg, Russia; c Faculty of Physics, M.V. Lomonosov Moscow State University, 119991, Moscow, Russia

**Keywords:** New langasites, Infrared and Raman spectra, *ab initio* calculations, Optimized crystal structures, Calculated frequencies and intensities of IR and Raman modes

## Abstract

In “Lattice dynamics and structure of the new langasites Ln_3_CrGe_3_Be_2_O_14_ (Ln = La, Pr, Nd): vibrational spectra and *ab initio* calculations” [1], experimental and calculated results on lattice dynamics of the recently discovered new compounds La_3_CrGe_3_Be_2_O_14_, Pr_3_CrGe_3_Be_2_O_14_, and Nd_3_CrGe_3_Be_2_O_14_ are reported. These compounds belong to the langasite series and constitute a new class of low-dimensional antiferromagnets. The data presented in this article includes IR diffuse transmission spectra of powder samples of Ln_3_CrGe_3_Be_2_O_14_ (Ln = La, Pr, Nd) registered at room temperature with a Bruker 125HR Fourier spectrometer, Raman spectra taken in the backscattering geometry (also at room temperature) with a triple monochromator using the line 514, 5 nm of an argon laser as an excitation, results of the DFT calculations with the B3LYP and PBE0 hybrid functionals on the optimized crystal structures, eigenfrequencies and eigenvectors of the normal vibrational modes. These data can be used to analyse electron-phonon interaction and multiferroic properties of the new langasites and to compare the lattice dynamics of different langasites. The dataset is available on mendeley data public repository at https://doi.org/10.17632/32grbb4p82.1.

**Table:** Specifications Table

Subject	Materials Science
Specific subject area	Electronic, Optical and Magnetic Materials
Type of data	Table
	Figure
	Text file
How data were acquired	IR spectra were collected in diffuse transmission mode with Bruker 125HR Fourier spectrometer, Raman spectra were collected in the backscattering geometry with a home-made triple monochromator using the line 514, 5 nm of an argon laser as an excitation.
	The CRYSTAL14 program designed for simulating periodic structures in the MO LCAO approximation was used for DFT *ab initio* calculations. Quasi-relativistic pseudopotentials ECP46MWB, ECP59MWB, and ECP60MWB with corresponding valence basis sets ECPnMWB were taken for La, Pr, and Nd. All-electron basis sets of TZVP type were used for Cr, Ge, Be, and O.
Data format	Raw
	Analyzed
Parameters for data collection	Spectra were collected on powder samples at room temperature.
	Calculations were performed within the framework of MO LCAO approach and the density functional theory, by using B3LYP and PBE0 hybrid functionals which takes into account both the local and nonlocal (in the Hartree–Fock formalism) exchange.
Description of data collection	Infrared diffuse transmission spectra were registered with a Bruker 125HR Fourier spectrometer equipped with a DTGS and a liquid-nitrogen-cooled MCT detectors. Raman spectra were collected in the backscattering geometry with a home-made triple monochromator (λ_excit_ = 514,5 nm).
	The frequencies and eigenvectors of the normal vibrational modes were obtained from *ab initio* calculations. First, the full geometry optimization (atomic positions and all unit-cell parameters) was carried out. Then, the phonon spectrum (at the Г- point) was calculated for the crystal structure corresponding to the minimum energy.
Data source location	Institute of Spectroscopy, Russian Academy of Sciences, Troitsk, Moscow,
	Russian Federation
	55.464596°N37.297538°E
Data accessibility	Repository name: Mendeley Data
	Data identification number: 32grbb4p82.1
	Direct URL to data: https://doi.org/10.17632/32grbb4p82.1
Related research article	N.N. Kuzmin et al., Lattice dynamics and structure of the new langasites Ln_3_CrGe_3_Be_2_O_14_ (Ln = La, Pr, Nd): vibrational spectra and *ab initio* calculations, Journal of Physics and Chemistry of Solids, In Press

**Table:** 

**Value of the Data** • These data can be used to compare the lattice dynamics of different langasites. • These data can be used by researchers working on vibrational and magnetoelastic properties of langasites. • These data can be used to analyse electron-phonon interaction and multiferroic properties of the new langasites.

## 1 Data description

The dataset includes 6 text files for our measured infrared (IR) and Raman spectra of Ln_3_CrGe_3_Be_2_O_14_ (Ln = La, Pr, Nd, raw data) [1]. These text files are named by rare-earth (RE) element symbol plus the method used to take the spectrum, e.g., Pr_IR.txt means an IR spectrum of Pr_3_CrGe_3_Be_2_O_14_. Each text file has two columns which correspond to wave number (unit: cm^−1^) and IR absorbance or Raman intensity (in arbitrary units). The same data are presented also as Excel files, e.g., Pr_IR.xlsx.

The data of *ab initio* calculations of optimized crystal structures is provided in 5 Excel tables. Table 1 provides the coordinates of atoms in the unit cell for the optimized structures of Ln_3_CrGe_3_Be_2_O_14_ (Ln = La, Pr, Nd), calculated with the B3LYP hybrid functional. Table 2 provides the interatomic distances for the optimized structures of Ln_3_CrGe_3_Be_2_O_14_ (Ln = La, Pr, Nd), calculated with the B3LYP hybrid functional. Table 3 provides the lattice constants for the optimized structures of Ln_3_CrGe_3_Be_2_O_14_ (Ln = La, Pr, Nd), calculated with the PBE0 hybrid functional. Table 4 provides the coordinates of atoms in the unit cell for the optimized structures of Ln_3_CrGe_3_Be_2_O_14_ (Ln = La, Pr, Nd), calculated with the PBE0 hybrid functional. Table 5 provides the interatomic distances for the optimized structures of Ln_3_CrGe_3_Be_2_O_14_ (Ln = La, Pr, Nd), calculated with the PBE0 hybrid functional. In Table 1, Table 2, Table 3, Table 4, Table 5, available experimental data are in square brackets.

**Table 1:** Calculated (B3LYP) coordinates of atoms in the unit cell of Ln_3_CrGe_3_Be_2_O_14_ (Ln = La, Pr, Nd). The experimental data for Ln_3_CrGe_3_Be_2_O_14_ [14] are shown in square brackets.

Ion	site	Ln = La			Ln = Pr			Ln = Nd		
		*x*/*a*	*y*/*b*	*z*/*c*	*x*/*a*	*y*/*b*	*z*/*c*	*x*/*a*	*y*/*b*	*z*/*c*
Ln	3*e*	0.42858 [0.42983(4)]	0.	0.	0.42797	0.	0.	0.42776	0.	0.
Cr	1*a*	0.	0.	0.	0.	0.	0.	0.	0.	0.
Ge	3*f*	0.74264 [0.74350(8)]	0.	0.5	0.74243	0	0.5	0.74235	0.	0.5
Be	2*d*	1/3	2/3	0.52202 [0.5260(10)]	1/3	2/3	0.52576	1/3	2/3	0.52735
O1	2*d*	1/3	2/3	0.20577 [0.1973(9)]	1/3	2/3	0.20729	1/3	2/3	0.20790
O2	6*g*	0.46708 [0.4671(4)]	0.30292 [0.3049(3)]	0.32707 [0.3251(5)]	0.46748	0.30543	0.32133	0.46770	0.30667	0.31882
O3	6*g*	0.22247 [0.2256(3)]	0.09505 [0.0966(3)]	0.75926 [0.7571(4)]	0.22352	0.09250	0.75799	0.22402	0.09133	0.75743

**Table 2:** Calculated (B3LYP) and experimentally determined [14] (in square brackets) M – O distances (Å) in the structure of Ln_3_CrGe_3_Be_2_O_14_ (Ln = La, Pr, Nd).

	Ln = La	Ln = Pr	Ln = Nd
R–polyhedron			
R–O1 × 2	2.637 [2.577(2)]	2.621	2.613
R–O2 × 2	2.520 [2.457(4)]	2.473	2.451
R–O2′ × 2	2.850 [2.816(3)]	2.827	2.818
R–O3 × 2	2.488 [2.450(3)]	2.449	2.431
(R–O)_av_	2.624 [2.575]	2.593	2.578
Cr–octahedron			
Cr–O3 × 6	1.987 [1.979(2)]	1.984	1.983
Ge– tetrahedron			
Ge–O2 × 2	1.784 [1.760(4)]	1.781	1.780
Ge–O3 × 2	1.774 [1.733(3)]	1.775	1.775
(Ge–O)_av_	1.779 [1.747]	1.778	1.778
Be–tetrahedron			
Be–O1	1.586 [1.622(6)]	1.589	1.590
Be–O2 × 3	1.698 [1.672(3)]	1.694	1.692
(Be–O)_av_	1.670 [1.660]	1.668	1.667

**Table 3:** Experimentally determined [14] and calculated (PBE0) lattice constants (Å) of Ln_3_CrGe_3_Be_2_O_14_.

Ln_3_CrGe_3_Be_2_O_14_		a	c
La_3_CrGe_3_Be_2_O_14_	Exp.	8.033(2)	4.934(2)
	Calc.	8.0622	4.9680
Pr_3_CrGe_3_Be_2_O_14_	Exp.	7.957(2)	4.904(2)
	Calc.	7.9968	4.9433
Nd_3_CrGe_3_Be_2_O_14_	Exp.	7.931(2)	4.894(2)
	Calc.	7.9683	4.9323

**Table 4:** Calculated (PBE0) and experimentally determined [14] (in square brackets) coordinates of atoms in the unit cell of Ln_3_CrGe_3_Be_2_O_14+_ (Ln = La, Pr, Nd).

Ion	site	Ln = La			Ln = Pr			Ln = Nd		
		*x*/*a*	*y*/*b*	*z*/*c*	*x*/*a*	*y*/*b*	*z*/*c*	*x*/*a*	*y*/*b*	*z*/*c*
Ln	3*e*	0.43071 [0.42983(4)]	0	0	0.43021	0	0	0.42976	0	0
Cr	1*a*	0	0	0	0	0	0	0	0	0
Ge	3*f*	0.74464 [0.74350(8)]	0	0.5	0.74435	0	0.5	0.74428	0	0.5
Be	2*d*	1/3	2/3	0.52203 [0.5260(10)]	1/3	2/3	0.52539	1/3	2/3	0.52713
O1	2*d*	1/3	2/3	0.20488 [0.1973(9)]	1/3	2/3	0.20613	1/3	2/3	0.20711
O2	6*g*	0.46553 [0.4671(4)]	0.30026 [0.3049(3)]	0.32701 [0.3251(5)]	0.46600	0.30279	0.32134	0.46618	0.30408	0.31883
O3	6*g*	0.22215 [0.2256(3)]	0.09317 [0.0966(3)]	0.75867 [0.7571(4)]	0.22329	0.09093	0.75757	0.22364	0.08958	0.75679

**Table 5:** Calculated (PBE0) and experimentally determined [14] (in square brackets) M – O distances (Å) in the structure of Ln_3_CrGe_3_Be_2_O_14_ (Ln = La, Pr, Nd).

	Ln = La	Ln = Pr	Ln = Nd
R–polyhedron			
R–O1 × 2	2.601 [2.577(2)]	2.584	2.578
R–O2 × 2	2.496 [2.457(4)]	2.449	2.429
R–O2′ × 2	2.811 [2.816(3)]	2.788	2.779
R–O3 × 2	2.468 [2.450(3)]	2.430	2.412
(R–O)_ср_	2.594 [2.575]	2.563	2.550
Cr–octahedron			
Cr–O3 × 6	1.966 [1.979(2)]	1.963	1.963
Ge– tetrahedron			
Ge–O2 × 2	1.769 [1.760(4)]	1.766	1.764
Ge–O3 × 2	1.756 [1.733(3)]	1.756	1.756
(Ge–O)_ср_	1.762 [1.747]	1.761	1.760
Be–tetrahedron			
Be–O1	1.576 [1.622(6)]	1.578	1.579
Be–O2 × 3	1.683 [1.672(3)]	1.678	1.677
(Be–O)_ср_	1.656 [1.660]	1.653	1.653

The dataset includes 3 text files for the calculated with the B3LYP hybrid functional frequencies of normal modes and their intensities in the IR and Raman spectra. These text files are named by RE element symbol plus the method to get the data, e.g., Pr_abinit.txt means the calculated data for Pr_3_CrGe_3_Be_2_O_14_. Each text file has four columns which correspond to the symmetry of the mode (irreducible representation), wave number (unit: cm^−1^), IR intensity, Raman intensity (arb. units). First, all A_1_ modes are listed, they are followed by the A_2_ and, then, E modes. The same data are presented also as Excel files, e.g., Pr_abinit.xlsx. Three Excel Tables, Table 6, Table 7, and Table 8**,** provide all calculated modes compared with those found from the measured spectra (analyzed data), in increasing order of their frequency for La_3_CrGe_3_Be_2_O_14_, Pr_3_CrGe_3_Be_2_O_14_, and Nd_3_CrGe_3_Be_2_O_14_, respectively. Mode symmetries are indicated.

**Table 6:** Experimentally determined [1] and calculated (B3LYP) frequencies in the Raman (R) and infrared (IR) spectra of La_3_CrGe_3_Be_2_O_14_.

Exp, R	Calculated			Exp, IR	Exp, R	Calculated				Exp, IR
	A1	E	A2			A1	E	A2		
R a m a n – a c t i v e					R a m a n – a c t i v e					
		I R – a c t i v e					I R – a c t i v e			
			88					396		400
108		105		100	406	396				
108		109			433		425			424
122	127				462		456			456
			130	133	488		482			
143		141						499		506
			156		544		532			551
159		164		167	568	561				
190		193		189				580		
	212				586	583				
			213	216	625		624			625
233		235					661			
		241		245			715			722
259		269		265	730		728			722
	284							733		741
			287		783		781			789
292		290		294	783	785				
292	292						809			
326		328						816		
			332			818				
351		343		344				825		821
378		376		384	836	839

**Table 7:** Experimentally determined [1] and calculated (B3LYP) frequencies in the Raman (R) and infrared (IR) spectra of Pr_3_CrGe_3_Be_2_O_14_.

Exp, R	Calculated			Exp, IR	Exp, R	Calculated			Exp, IR
	A1	E	A2			A1	E	A2	
R a m a n – a c t i v e					R a m a n – a c t i v e				
		I R – a c t i v e					I R – a c t i v e		
			83	89				398	404
		96		89	409	401			
108		108			435		431		430
123	127				462		459		462
			131	135	489		485		
145		141						503	509
			150		548		538		556
161		164		166	575	565			
193		194		188				583	
	215				587	587			
			216		631		632		633
235		235					668		
		243		245			721		728
260		265		263				734,5	743
	284				732		734,8		743
			286		783		785,6		794
295		294		297	783	785,7			
295	297						813		
		327				818			
			337					821	823
355		348		352				823	823
382		378		385	836	843

**Table 8:** Experimentally determined [1] and calculated (B3LYP) frequencies in the Raman (R) and infrared (IR) spectra of Nd_3_CrGe_3_Be_2_O_14_.

Exp, R	Calculated			Exp, IR	Exp, R	Calculated			Exp, IR
	A1	E	A2			A1	E	A2	
R a m a n – a c t i v e					R a m a n – a c t i v e				
		I R – a c t i v e					I R – a c t i v e		
			80					400	405
		92		90	410	404			
108		108			436		434		435
123	127				463		461		463
			131	138	490		486		
146		141						505	512
			148		549		540		560
160		165		166	572	569			
191		194		191				585	
	217				589	590			
			218		626		635		634
236		236					671		
		244		247			723		731
261		264		265	734			735	746
	284						738		
			286		784	786			794
296		295		301	784		787		794
	301						814		
330		327				817			
			340					820	
356		351		354				826	823
382		380		385	838	845			

The data on calculated displacements of different atoms in normal crystal modes of different frequencies for Ln_3_CrGe_3_Be_2_O_14_ (Ln = La, Pr, Nd) is provided in 3 text files named, e.g., Pr_displ.txt. Each text file has eight columns. The first column correspond to the mode frequency (unit: cm^−1^), the columns 2–8 correspond to the displacements (unit: Å) of the following atoms: Ln, Cr, Ge, Be, O1, O2, O3. The same data are presented also as Excel tables with 8 columns, named, e.g., Pr_displ.xlsx.

**Fig. 1:**
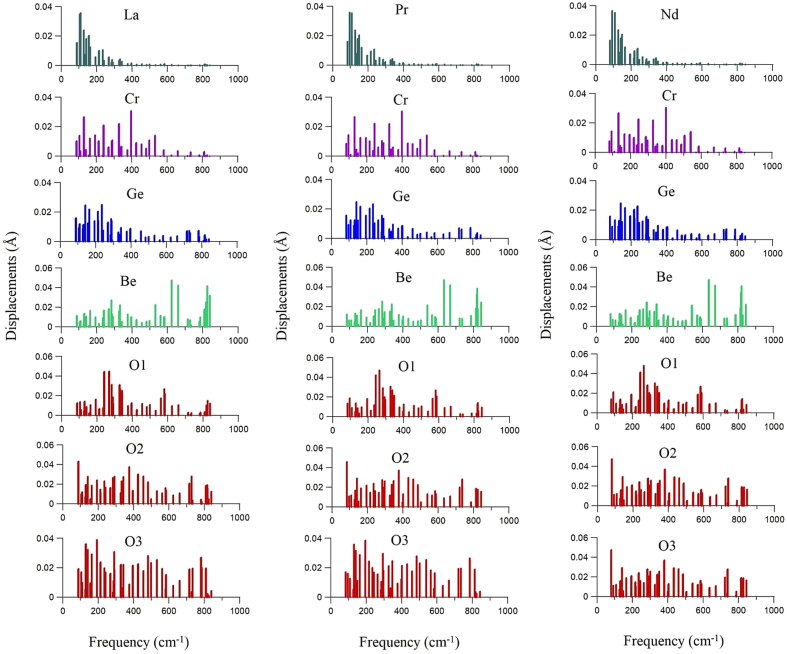
Displacements of different atoms of La_3_CrGe_3_Be_2_O_14_ in normal crystal modes of different frequencies.

Fig. 1 depicts these displacements for all three title compounds, namely, La_3_CrGe_3_Be_2_O_14_, Pr_3_CrGe_3_Be_2_O_14_, and Nd_3_CrGe_3_Be_2_O_14_. It is given as the eps and opj files, Figure 1.eps and Figure 1.opj, respectively. The table in text (and Excel) format Figure 1_table.txt (and Figure 1_table.xlxs) provides the data necessary to create Figure 1.

## 2 Experimental design, materials, and methods

The main information on the samples and experimental equipment used to take the spectra, as well as on the calculation methods is presented in Ref. [1]. Powder samples of the studied compounds La_3_CrGe_3_Be_2_O_14_, Pr_3_CrGe_3_Be_2_O_14_, and Nd_3_CrGe_3_Be_2_O_14_ were synthesized by a high-temperature solid-state reaction from high-purity La_2_O_3_, Pr_2_O_3_, Nd_2_O_3_ and GeO_2_, Cr_2_O_3_ (reagent grade), and BeO (99.54%). Stoichiometric amounts of oxides were thoroughly ground together, pressed into pellets, placed on a Pt substrate and sintered in air for 5 h at 1350^о^С (the Nd and Pr compounds) and at 1325^о^С (the La compound). To reduce the loss of GeO_2_ due to evaporation, the pressed samples were encapsulated in the original powdered charges. The phase composition of sintering products was studied by X-ray diffraction using a diffractometer STOE STADI_MP in a transmission mode (Cu*K*_α1_ radiation). The spасе group *P*321 was confirmed for all samples.

The infrared diffuse transmission and Raman scattering spectra of Ln_3_CrGe_3_Be_2_O_14_ (Ln = La, Pr, Nd) powder samples were measured at room temperature. Powders of Ln_3_CrGe_3_Be_2_O_14_ were mixed with optical-grade KBr powder and pressed into pellets. Far-infrared diffuse transmission spectra were registered in the spectral region 50–1200 cm^−1^ at a resolution 2 cm^−1^ using a Fourier spectrometer Bruker IFS 125HR and a DTGS and a liquid-nitrogen-cooled MCT detectors. Raman spectra were taken in the backscattering geometry at a resolution 3 cm^−1^ with a home-made triple monochromator using the line 514, 5 nm of an argon laser as an excitation.

*Ab initio* calculations of phonon frequencies and intensities of the infrared- and Raman-active modes of La_3_CrGe_3_Be_2_O_14_, Pr_3_CrGe_3_Be_2_O_14_, and Nd_3_CrGe_3_Be_2_O_14_ were performed in a framework of the density functional theory (DFT) with the hybrid functional B3LYP [2], which takes into account both local and nonlocal (in the Hartree-Fock formalism) exchange. The sequence of calculations was as follows. The optimization of the crystal structure was carried out first. After that, the phonon spectrum was calculated for the crystal structure corresponding to the minimum energy. The CRYSTAL14 program [3] designed for simulating periodic structures in the MO LCAO approximation was used for calculations. Quasi-relativistic pseudopotentials ECP*4*6MWB, ECP*5*9MWB, and ECP*6*0MWB [4,5] with corresponding valence basis sets ECP*n*MWB [6] were taken for La, Pr, and Nd. All-electron basis sets of TZVP type were used for Cr, Ge, Be, and O [7]. These basis sets are available at the CRYSTAL website. The reciprocal space sampling was performed by Monkhorst-Pack mesh. The algorithm of calculation of the two-electron Coulomb and exchange integrals is given in Ref. [8]. The tolerance of self-consistently solving of the system of Kohn-Sham equations was 10^−9^. The phonon spectrum was calculated in the harmonic approximation. In the Hessian matrix, the first (second) derivatives were calculated analytically (numerically). To perform numerical calculations of the second derivatives, the atom was displaced from the equilibrium position by 0.003 Å [8].

We used the Born charges when calculating Raman and infrared intensities in the CRYSTAL code [9]. Electric dipole properties were calculated using the periodic coupled-perturbed Hartree-Fock (CPHF) or Kohn-Sham (CPKS) approach [[10], [11], [12]].

The Plaсzek approximation was used to calculate the intensity of the Raman modes at a non-resonant excitation [11]. For an oriented single crystal, the intensity associated with the mode $\omega_{k}$ is [3]:

(1) $$I_{ij}^{k}\propto C{\left(\alpha_{ij}^{k}\right)}^{2}\text{,}$$

where $\alpha_{ij}^{k}\;$ is an element of the Raman tensor, i,j=x,y,z. The value C in (1) is defined by the laser frequency $\omega_{L}$ and the temperature *T* dependence as follows:

(2) $$C∼\frac{1+n\left(\omega_{k}\right)}{30\omega_{k}}{\left(\omega_{L}-\omega_{k}\right)}^{4}\text{,}$$

where

(3) $$1+n\left(\omega_{k}\right)={\left[1-\exp\left(-\frac{\hbar \omega_{k}}{k_{B}T}\right)\right]}^{-1}\text{,}$$

$n\left(\omega_{k}\right)$ being the Bose occupation factor.

The simulation of the intensity of Raman modes for powder sample has been done by computing integrals over all possible orientations of ideal bulk crystal. These integrals can be reduced to three rotational invariants [13]:

(4) $$G_{k}^{\left(0\right)}=\frac{1}{3}{\left(\alpha_{xx}^{k}+\;\alpha_{yy}^{k}+\;\alpha_{zz}^{k}\right)}^{2}$$

(5) $$G_{k}^{\left(1\right)}=\frac{1}{2}\left[{\left(\alpha_{xy}^{k}-\;\alpha_{yx}^{k}\right)}^{2}+{\left(\alpha_{xz}^{k}-\;\alpha_{zx}^{k}\right)}^{2}\;+\;{\left(\alpha_{zy}^{k}-\;\alpha_{yz}^{k}\right)}^{2}\right]$$

(6) $$G_{k}^{\left(2\right)}=\frac{1}{2}\left[{\left(\alpha_{xy}^{k}+\alpha_{yx}^{k}\right)}^{2}+{\left(\alpha_{xz}^{k}+\alpha_{zx}^{k}\right)}^{2}+{\left(\alpha_{zy}^{k}+\alpha_{yz}^{k}\right)}^{2}\right]+\frac{1}{3}\left[{\left(\alpha_{xx}^{k}-\alpha_{yy}^{k}\right)}^{2}+{\left(\alpha_{xx}^{k}-\alpha_{zz}^{k}\right)}^{2}+{\left(\alpha_{yy}^{k}-\alpha_{zz}^{k}\right)}^{2}\right]$$

The intensity for the powder sample can be calculated as [14]:

(7) $$I_{tot,k}^{powder}=I_{∥,k}^{powder}+I_{⊥,k}^{powder}\text{,}$$

where

(8) $$I_{∥,k}^{powder}=C\left(10G_{k}^{\left(0\right)}+4G_{k}^{\left(2\right)}\right)$$

(9) $$I_{⊥,k}^{powder}=C\left(5G_{k}^{\left(1\right)}+3G_{k}^{\left(2\right)}\right)$$

and C is given by Eq. (2).

The infrared intensity of the *p*-th mode can be written as [3]:

(10) $$I_{p}\;=\;\frac{\pi }{3}\frac{N_{A}}{c^{2}}d_{p}{\left|{\vec{Z}}_{p}\right|}^{2}\text{,}$$

where $N_{A}$ is the Avogadro's number, c is the speed of light, $d_{p}$ is the degeneracy of the mode, ${\vec{Z}}_{p}$ is the mass-weighted Born eﬀective charge vector of the mode. The infrared intensity was calculated assuming an isotropic response.

The high-spin (S = 3/2) state of the Cr^3+^ ions was set in the calculations. At the simulation, magnetic moments of chromium ions were codirected (along the *z* axis), hereby the ferromagnetic state was simulated. In this work, we consecutively calculate the crystal structure and, then, the phonon spectrum. The initial structural data were taken from Ref. [14].

When choosing a functional, calculations with the hybrid functional PBE0 [15] were also performed.
